# Integrative cBioPortal Analysis Revealed Molecular Mechanisms That Regulate EGFR-PI3K-AKT-mTOR Pathway in Diffuse Gliomas of the Brain

**DOI:** 10.3390/cancers13133247

**Published:** 2021-06-29

**Authors:** Petar Brlek, Anja Kafka, Anja Bukovac, Nives Pećina-Šlaus

**Affiliations:** 1 Laboratory of Neurooncology, Croatian Institute for Brain Research, School of Medicine, University of Zagreb, 10000 Zagreb, Croatia; anja.kafka@mef.hr (A.K.); anja.bukovac@mef.hr (A.B.); 2 Department of Biology, School of Medicine, University of Zagreb, 10000 Zagreb, Croatia

**Keywords:** astrocytoma, glioblastoma, large-scale analysis, AKT, CHUK, GSK3β, PIK3AP1, PTEN, EGFR, cBioPortal

## Abstract

**Simple Summary:**

The current classification of central nervous system tumors has incorporated molecular changes that have clarified biological behavior and categorized gliomas into different types and malignancy grades. The most malignant type—glioblastoma, represents one of the most therapeutically challenging tumors, with a median survival of only 12–14 months despite trimodal therapy. In our integrative large-scale study, we used genomics, transcriptomics, epigenomics, and proteomics to investigate and make sense of the molecular changes that activate or inhibit the EGFR-PI3K-AKT-mTOR signaling pathway. Different pathohistological types of diffuse brain gliomas harbored distinct changes. A better understanding of signaling pathway regulation helps to the discovery of new targets for glioma therapies. Our results have potential for diagnostics improvement and tailored therapies.

**Abstract:**

Diffuse gliomas are a heterogeneous group of tumors with aggressive biological behavior and a lack of effective treatment methods. Despite new molecular findings, the differences between pathohistological types still require better understanding. In this in silico analysis, we investigated *AKT1*, *AKT2*, *AKT3*, *CHUK*, *GSK3β*, *EGFR*, *PTEN*, and *PIK3AP1* as participants of EGFR-PI3K-AKT-mTOR signaling using data from the publicly available cBioPortal platform. Integrative large-scale analyses investigated changes in copy number aberrations (CNA), methylation, mRNA transcription and protein expression within 751 samples of diffuse astrocytomas, anaplastic astrocytomas and glioblastomas. The study showed a significant percentage of CNA in *PTEN* (76%), *PIK3AP1* and *CHUK* (75% each), *EGFR* (74%), *AKT2* (39%), *AKT1* (32%), *AKT3* (19%) and *GSK3β* (18%) in the total sample. Comprehensive statistical analyses show how genomics and epigenomics affect the expression of examined genes differently across various pathohistological types and grades, suggesting that genes *AKT3*, *CHUK* and *PTEN* behave like tumor suppressors, while *AKT1*, *AKT2*, *EGFR*, and *PIK3AP1* show oncogenic behavior and are involved in enhanced activity of the EGFR-PI3K-AKT-mTOR signaling pathway. Our findings contribute to the knowledge of the molecular differences between pathohistological types and ultimately offer the possibility of new treatment targets and personalized therapies in patients with diffuse gliomas.

## 1. Introduction

Gliomas are a heterogeneous group of central nervous system tumors that still lack efficient treatment methods regardless of medical progress [1]. Despite multimodal treatment, consisting of surgical procedures, radiotherapy, and chemotherapy, the median survival for glioblastoma patients is only 12–14 months [2,3]. Recent studies of the molecular profile of gliomas show the complex dynamics of tumor progression and explain the evolution of mutations of this biologically aggressive group of tumors [4]. Recent findings in neurooncology have led to changes in the WHO (World Health Organization) classification from 2016 of tumors of the central nervous system, which incorporated both phenotypic and molecular parameters in the division of glial tumors. The latest classification groups all diffuse gliomas, whether astrocytic or not, into one category [5] based on mitotic activity, diffuse growth pattern, and the mutational status of the *IDH1* and *IDH2* genes, together with several other molecular signatures. New classification improved the prognosis of diffuse glioma. For example, patients with grade III gliomas carrying a 1p/19q co-deletion have a better prognosis than IDH wild-type grade II glioma [5]. Our present investigation focuses on a detailed analysis of the genetic and protein changes, but also epigenetic and transcriptional levels that aggregate complex regulatory mechanisms which could offer potential molecular prognostic and therapeutic targets. The new classification groups tumors that share the same prognostic markers and potentially allows for common treatment of molecularly similar entities [5]. Despite new knowledge that has united diffuse gliomas, the molecular differences between individual pathohistological types and grades are still insufficiently explained [6]. One of the reasons why we focused our interest on EGFR-PI3K-AKT-mTOR signaling pathway is that our previous investigation, using Array Comparative Genomic Hybridization (aCGH) and bioinformatics utilizing a Bioconductor package, Genomic Identification of Significant Targets in Cancer (GISTIC) 2.0.23 and DAVID software, identified main actors of the PI3K-AKT pathway activated in human astrocytomas and suggested that DNA copy number alterations play important roles in its etiology and progression [7]. Furthermore, the AKT signaling pathway mediates cell regulation, cell proliferation, cell cycle, and carbohydrate metabolism by further phosphorylation of GSK3β (glycogen synthase kinase 3 beta), Bad (bcl-2 bound death promoter), caspase-9, NF-κB (nuclear factor κB), mTOR (target molecule of rapamycin in mammals) and p21 protein [8,9,10,11,12,13]. Since recent studies have revealed the essential role of EGFR-PI3K-AKT-mTOR signaling in human tumors [14], we were interested in the molecular characteristics of this pathway across the three pathohistological types and grades of diffuse brain gliomas. Additionally, we focused on *CHUK* and *PIK3AP1* genes, which are important for regulating the PI3K-AKT-mTOR pathway. Recent studies showed an essential role of *CHUK* protein product (IKK) in glioma tumorigenesis [15], while *PIK3AP1* acts as a negative regulator of toll-like receptor-induced inflammation. The roles of both *CHUK* and *PIK3AP1* in various tumors are still insufficiently investigated [16,17]. Although several studies have indicated the important role of the PI3K-AKT-mTOR pathway in brain tumors, our study aim to provide a unique and comprehensive analysis of multi-omics data that may reveal a novel molecular background of diffuse brain gliomas. In addition, *PIK3AP1* and *CHUK* genes have not been investigated so far. Furthermore, specific molecules within this signaling pathway have been untested at the epigenetic and transcriptional levels. A need for a better understanding of the function and mechanisms of PI3K-AKT-mTOR signal transduction may eventually provide advanced diagnostic and treatment options for primary brain neoplasms [9,18]. This study aims to understand the significance of the EGFR-PI3K-AKT-mTOR signaling pathway and reveal differences between *AKT1*, *AKT2*, *AKT3*, *CHUK*, *GSK3β*, *EGFR*, *PTEN*, and *PIK3AP1* roles within different grades and types of diffuse brain gliomas.

## 2. Materials and Methods

### 2.1. cBioPortal Database and Bioinformatics Methods

Integrative analysis of diffuse brain gliomas was performed using cBioPortal (https://www.cbioportal.org/, accessed on 16 April 2021)—a publicly available database for tumor genomics and transcriptomics [19]. This database enables large-scale data processing, statistical analysis, and graphical review of tumor changes from gene to protein level [20]. We based our study on data generated by the TCGA research network (The Cancer Genome Atlas, https://www.cancer.gov/tcga, accessed on 16 April 2021), containing a total of more than 400 terabytes of raw data for 33 different tumor types requiring analysis [21]. Using the cBioPortal database, we created a virtual study using a combination of data provided by two previous large-scale studies: Brain Lower Grade Glioma (TCGA Firehose Legacy) and Glioblastoma Multiforme (TCGA Firehose Legacy). The samples from original individual publications [22,23,24,25,26,27,28] of TCGA data are a subset of our Firehose Legacy dataset from which the data were collected within cBioPortal. All TCGA Firehose Legacy data are publicly available at https://gdac.broadinstitute.org/ (accessed on 16 April 2021) and are updated every three months to contain the most complete and accurate data. We selected samples that held data for the following parameters: sex and age of the patient at diagnosis, a patient without multiple samples, a patient with complete genetic analysis, selected pathohistological types and grades of diffuse gliomas (diffuse astrocytoma (DA, grade II), anaplastic astrocytoma (AA, grade III), glioblastoma multiforme (GBM, grade IV)). In the created virtual study using the OQL programming language (Onco Query Language), genes were queried and the parameters selected for the analysis were as follows: changes in the number of copies of selected genes (CNA—copy number alteration), transcription levels (mRNA), methylation patterns and protein expression levels. Analysis of CNA was profiled on 751 samples, of which 62 DA, 129 AA and 560 GBM. mRNA expression data were available for 62 DA, 129 AA, and 140 GBM. To compare protein expression for each pathohistological grade, cBioPortal contained protein expression data for 348 gliomas, of which 46 DA, 99 AA, and 213 GBM. Protein expression data were lacking for the CHUK and PIK3AP1 proteins in all three types of gliomas examined. To compare the methylation of the relevant genes between individual pathohistological type and grade, 62 DA, 129 AA, and 123 GBM had data available for the analysis. Methylation data for the *AKT2* gene were missing for two AA. However, methylation data for the *PIK3AP1* gene were not available for the GBM group. All cBioPortal data have the same clinical criteria and equally processed and normalized data, enabling comparative analysis of samples between different studies. After creating the virtual study, CNA, methylation data, mRNA expression, and protein expression of selected genes were downloaded and examined like a classical study. Graphs of CNA were made in Excel 2016 (Microsoft), while graphs of methylation and mRNA expression were made using the software package IBM SPSS Statistics 23.0 (SPSS, Chicago, IL, USA). Data downloaded from a publicly available cBioPortal database does not require ethical approval. All patients whose samples were used in this analysis signed informed consent. Our virtual study, created on 16 April 2021, has data for download available at https://www.cbioportal.org/study?id=609e74e2e4b015b63e9eb2d6 (Supplementary Materials). The current version of the publicly available cBioPortal uses hg19/GRCh37 as a reference to the human genome.

### 2.2. Data Processing

A protocol for processing and normalizing TCGA data was based on a study by Hoadley et al. [22], and additional documentation is publicly available on the Broad Institute Firehose website (https://gdac.broadinstitute.org/ accessed on 16 April 2021).

RNA and DNA were obtained from tumor and adjoining normal tissue specimens using an adjustment of the DNA/RNA AllPrep kit (QIAGEN). Pathologists systematically reviewed samples to verify the histopathologic diagnosis and any irregularity in histology, applying the criteria of the most up-to-date edition of the WHO Classification of Tumors relevant to each tumor type. Any non-concordant diagnoses amongst the pathologists were re-reviewed, and a decision was achieved after examination [22].

Copy-number data were generated on Affymetrix SNP 6.0 arrays using regular protocols from the Genome Analysis Platform of the Broad Institute [22]. CNA are continuous values of the number of copies of a gene obtained as the difference between the number of copies of a tumor gene and the reference. Normalized continuous CNA values were processed within cBioPortal using the Genomic Identification of Significant Targets in Cancer (GISTIC 2.0) algorithm [29], and continuous values lower than −2.0 were listed as homozygous deletions (HOMDEL), while values between −2.0 and −1.0 were listed as hemizygous deletions (HETLOSS). In contrast, values between 1.0 and 2.0 were declared amplifications (GAIN), and more than 2.0 multiple amplifications (AMP). In addition, samples whose continuous CNA value was between −1.0 and 1.0 were declared diploid samples (DIPLOID), meaning they have no changes in the number of gene copies. Since the results obtained by the GISTIC 2.0 algorithm correlate well with the actual amplifications and deletions of one or more alleles, in this paper we used the following terminology: homozygous deletions (loss of both alleles), hemizygous deletions (loss of one allele), diploid samples (samples with two alleles—without CNA), amplifications (existence of 3 copies of alleles) and multiple amplifications (existence of 4 or more copies of alleles).

To obtain mRNA expression levels, next-generation sequencing from RNASeq V2 RSEM (RNA-seq by Experimentation Maximization) dataset was downloaded [30]. RSEM determines the total RNA transcript [20]. The expression data assigned from Illumina were batch-corrected to correct platform variations between the GAII and HiSeq Illumina sequencers. Additional corrections were made for various sequencing centers (British Columbia Cancer Agency and The University of North Carolina) [22]. More precisely, the RNASeq V2 data in cBioPortal matches the rsem.genes.normalized_results file from TCGA. cBioPortal mRNA expression data are calculated as relative expression of a specific gene in a tumor sample to the gene’s expression distribution in a reference (all samples that are diploid for the gene in question) population of samples [20].

Illumina Infinium DNA methylation arrays were applied to collect DNA methylation profiles of three tumor types and histologically normal tumor-adjacent tissue. All our samples were profiled on the same methylation array (HumanMethylation450 (HM450) Infinium array) [31]. The HM450 array relies on a chemical reaction that converts methylated cytosine to thymine by treatment with sodium bisulfite. Each CpG site targets Infinium I (one probe corresponds to the methylated version and the other corresponds to the non-methylated version of the analogous CpG site) and Infinium II probes (one probe for target CpG loci and differently labeled nucleotides to determine methylation status). Due to the variation in intensity (and β-values) displayed by Infinium I and Infinium II probes, a quantile normalization of the data was made to produce a similar distribution of β-values between Infinium I and II probes [32]. The level of methylation at the associated CpG site is measured as a beta value indicating the ratio between the intensity of the methylated series and the total intensity of the series, which falls between 0 (weakest methylation) and 1 (strongest methylation) [22]. Normalization of data was done using different internal controls that are present on the HumanMethylation450 BeadChip. Using the HM450 probe sequence information implemented in the manufacturer’s manifest, the probes were re-mapped using the reference genome. These coordinates were then used to link transcripts to GENCODE v22, the associated CpG island, and the distance of the CpG site from each of these traits. Illumina annotates CpG positions on the chip relative to RefSeq gene traits. RefSeq genes are covered across gene regions (promoter region, 5′UTR, first exon, gene body, 3′UTR) and extended to CpG island regions. [32]. Methylation data were not additionally processed or normalized within the cBioPortal.

Protein expression data were assessed using the reverse-phase protein array (RPPA) platform that provided expression levels for proteins and phosphoproteins. During the data normalization process, expression data (RPPA) for protein were batch effects-corrected and median-centered in both directions [22]. RPPA (reverse-phase protein array) applies panels of high-specificity antibodies to determine total protein expression in biological and clinical research. The method allows well-defined and sensitive quantification of a significant number of samples. Within cBioPortal, the protein data were additionally processed and normalized with the calculation of the z-scores and converted on the log scale [20].

### 2.3. Statistical Analysis

All samples were statistically analyzed according to the following variables: pathohistological diagnosis, sex, age, CNA type, mRNA expression, methylation level, and protein expression for each gene (*AKT1*, *AKT2*, *AKT3*, *CHUK*, *GSK3β*, *EGFR*, *PTEN* and *PIK3AP1*). Statistical analysis of the obtained data was performed in the software package IBM SPSS Statistics 23.0 (SPSS, Chicago, IL, USA), with a significance level of *p* < 0.05. Continuous data values were used for the analysis of the CNA, while values obtained by the GISTIC algorithm were used for the graphical representation. For all other parameters (mRNA, methylation, protein expression), normalized continuous values were used for graphical presentation and statistical analysis. The normality of the distribution of individual parameters within the groups was tested using the Kolmogorov–Smirnov and Shapiro–Wilk test of normality (a low significant value (*p* < 0.05) indicates that the distribution differs significantly from the average). Depending on the results of those tests parametric or nonparametric statistical tests were used accordingly. The Kruskal–Wallis test tested differences in measured values of CNA, mRNA, methylation, and protein expression between the three pathohistological types of gliomas. The Mann–Whitney test was employed to compare individual groups. The Kruskal–Wallis and Mann–Whitney tests were also used to compare the age of patients in individual grades of diffuse gliomas. Correlations between CNA and mRNA expression, methylation and mRNA expression, and protein and mRNA expression were tested by the Spearman’s test.

## 3. Results

### 3.1. Demographic Data of Examined Diffuse Brain Gliomas

Diffuse glioma samples were collected from 446 males and 305 females. The examined patients’ age ranged from 10 to 89 years, while the median age at the time of diagnosis was 55 years. The median age for men was 56 years, and for women 54 years. Our study included 751 samples of diffuse brain gliomas of various grades of malignancy, of which 62 were diffuse astrocytomas (DA), 129 anaplastic astrocytomas (AA), and 560 glioblastomas multiforme (GBM) (Table 1).

The Kruskal–Wallis test showed a statistically significant difference in the age of the disease onset within the different pathohistological types of diffuse gliomas (*p* < 0.001). Using the Mann–Whitney test, we confirmed significant difference between the median age and pathohistological type of brain glioma (Table 1).

### 3.2. Changes in the Copy Number (CNA) of Examined Genes

In silico analysis included the following genes: *AKT1*, *AKT2*, *AKT3*, *CHUK*, *GSK3β*, *EGFR*, *PTEN*, and *PIK3AP1*. Analysis of CNA in a total sample of diffuse brain gliomas showed that the highest total number of CNA was confined to the GBM group, while the lowest CNA of all tested genes was found in the DA group. This result shows the accumulation of gene aberrations during glioma progression and is consistent with the biological behavior of the most aggressive type—GBM. Further analysis showed that GBM harbored the highest number of CNA in genes *CHUK* (88%), *EGFR* (89%), *PIK3AP1* (89%), and *PTEN* (89%). Lower grade gliomas, the AA group, harbored the highest number of CNA in genes *PTEN* (50%), *PIK3AP1* (49%), and *CHUK* (49%), while the DA group carried the highest number of CNA in genes *AKT1* (15%), *AKT2* (11%), and *GSK3β* (11%). We found that aberrations in the lowest malignancy type (DA) affects *AKT1*, *AKT2* and *GSK3β* genes with a similar frequency, while a different set of genes (*CHUK*, *EGFR*, *PIK3AP1*, and *PTEN)* were highly affected in GBM (Table 2). This suggests that the *AKT1*, *AKT2*, and *GSK3β* genes may be involved in the early stages of diffuse glioma formation, while changes in the *CHUK*, *EGFR*, *PIK3AP1* and *PTEN* genes accumulate during progression. The Kruskal–Wallis test revealed a significant difference in the relative continuous number of CNA between individual pathohistological types for genes *AKT2* (*p* < 0.001), *AKT3* (*p* < 0.001), *CHUK* (*p* < 0.001), *EGFR* (*p* < 0.001), *PIK3AP1* (*p* < 0.001) and *PTEN* (*p* < 0.001). A detailed analysis between individual pathohistological types was performed by the Mann–Whitney test and is presented in the following subheadings.

### 3.3. CNA of AKT1, AKT2 and AKT3—the Core of the Signaling Pathway

Hemizygous deletions (loss of one allele) were the most common CNA found for the *AKT1* gene, comprising 26% of total samples. Hemizygous deletions were found in 151 (27%) glioblastomas and were significantly more frequent than amplifications (the existence of three copies of alleles). The AA and DA groups harbored 26% and 15% of hemizygous deletions, respectively. Amplifications were found in 6% of GBM and 2% of AA. Significant differences between pathohistological types were not established, although it was evident that the number of *AKT1* deletions were more frequent in GBM. Contrary to *AKT1*, the analysis of the *AKT2* gene revealed that the most common CNAs in the total sample were amplifications (26%). They were found in 33% of GBM and 10% of AA, while DA did not harbor amplifications. Hemizygous deletions of *AKT2* were distributed to 10% of GBM, 20% of AA and 6% of DA. The results of the Mann–Whitney test showed a statistically significant difference in pathohistological types (Table 2). These results show that GBM was significantly more affected by the amplification of *AKT2* compared to AA and DA. Analysis of the *AKT3* gene revealed behavior similar to that of *AKT2*, showing that amplification was again the most commonly found CNA, adding up to 13% of the total sample, of which 15% were associated with GBM, 10% with AA and 2% with DA. Other CNAs for all three genes were recorded in traces. The Mann–Whitney test showed significant differences in the continuous value of the *AKT3* gene CNA between DA and GBM (*p* < 0.001) and between AA and GBM (*p* < 0.001). These results confirm a significantly higher prevalence of amplification in grade IV. The distribution of CNA for *AKT1*, *AKT2* and *AKT3* within each pathohistological type and grade is shown in Table 2.

### 3.4. CNA of CHUK, GSK3β and PTEN—Regulators of PI3K-AKT-mTOR Pathway

Analysis of *CHUK* showed that in the total sample examined, hemizygous deletions were the most common CNA of this gene with 557 cases (74%). They were distributed to 491 samples (88%) of GBM, 47% of AA and 8% of DA. The Mann–Whitney test showed significant differences in the continuous value of the CNA of the *CHUK* gene between pathohistological types (Table 2). *CHUK* was significantly more affected by deletions at higher grades. Contrary to this finding, changes in *GSK3β* were not frequent. A total of 135 samples (18%) had CNA (Table 2), of which the most common were amplifications, found in a total of 65 (9%) samples. These changes affected 59 (11%) GBM. However, hemizygous deletions of this gene were also present in 64 (9%) cases of the total sample. The glioblastoma group harbored 53 (9%) hemizygous deletions, while the DA group showed homozygous deletions (loss of both alleles) in 5% of samples and no other CNAs were recorded. The result of CNA analysis of the *PTEN* gene demonstrated that hemizygous deletions were the most common aberration, occurring in a total of 506 (67%) samples. These changes were distributed to 79% of GBM, 46% of AA and 8% of DA. Homozygous deletions were also present in 10% of GBM, 4% of AA and 0% of DA. All other CNAs were recorded in traces. The Mann–Whitney test showed a significant difference in the continuous value of the CNA between pathohistological types. These results confirmed that *PTEN* suffered significant deletions at higher grades. *CHUK*, *GSK3β* and *PTEN* distribution of CNA within each pathohistological type and grade are shown in Table 2.

### 3.5. CNA of EGFR and PIK3AP1—Activators of PI3K-AKT-mTOR Pathway

The *EGFR* gene was frequently affected by CNA—altogether in 554 (74%) glioma samples. Amplification was the most common CNA, and was found in 275 patients (37%) out of our total sample. Amplifications were distributed to 44% of GBM, 22% of AA and 5% of DA. Multiple amplifications (four or more copies of alleles) were also common in a total of 272 (36%) samples, of which 247 cases (44%) were in GBM, while 19% of AA and 2% of DA showed this aberration. The Mann–Whitney test showed a significant difference in the continuous values of the CNA of the *EGFR* gene between pathohistological types (Table 2). These results indicate that amplifications and multiple amplifications of this gene progressively increased and were significantly more associated with higher grades, mainly grade IV. Analysis of the *PIK3AP1* gene revealed that hemizygous deletions were very common in the examined sample, with 88% altered in GMB, 47% in AA and 8% in DA. Hemizygous deletions were found in a total of 556 (74%) samples. The Mann–Whitney test showed a significant difference in the continuous value of the CNA between pathohistological types. The higher grades, GBM in particular, were more strongly affected by CNA, this time with deletions of this gene. *EGFR* and *PIK3AP1* distribution of CNA within each pathohistological type are shown in Table 2.

### 3.6. mRNA Expression Levels of the EGFR-PI3K-AKT-mTOR Pathway Participants

**Figure 1:**
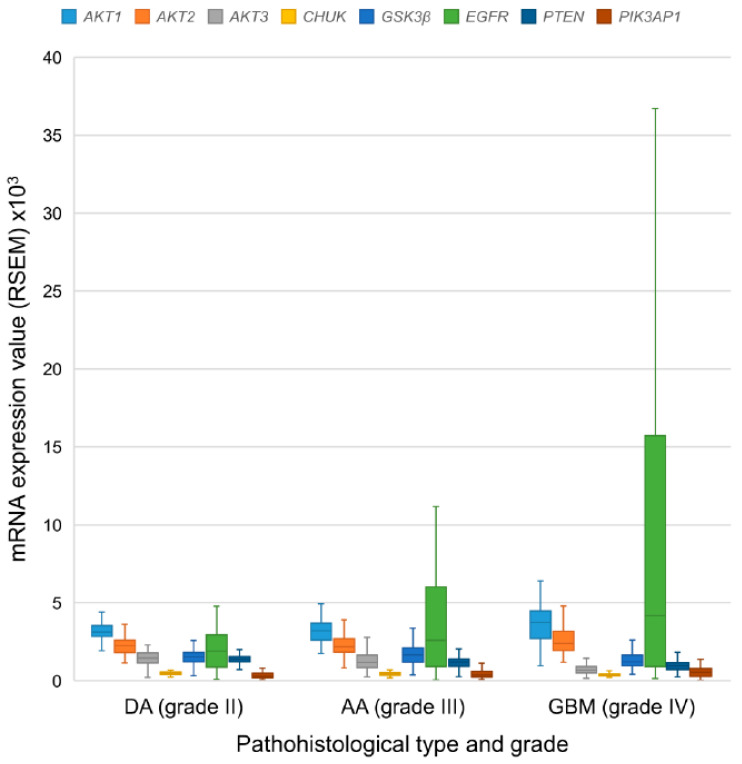
Distribution of mRNA expression obtained by the RSEM method of the examined genes according to the pathohistological type and grade of diffuse gliomas. DA—diffuse astrocytoma; AA—anaplastic astrocytoma; GBM—glioblastoma multiforme.

*AKT1* gene transcripts showed the highest level of mRNA expression in DA, while in AA and GBM the highest mRNA expression was associated with the *EGFR* gene. *PIK3AP1* showed the lowest mRNA expression in DA, while in AA and GBM the lowest levels were associated with *CHUK* gene. Table 3 and Figure 1 show the mRNA expression levels for each gene. There was significant difference in mRNA expression of all examined genes between individual pathohistological types (Kruskal–Wallis test *p* < 0.001 for *AKT1*, *AKT3*, *CHUK*, *GSK3β*, *PTEN*, and *PIK3AP1*; *p* = 0.025 for *AKT2*; *p* = 0.003 for *EGFR*). The Mann–Whitney test showed that there were significantly lower transcript levels of *AKT3* (*p* = 0.019), *CHUK* (*p* = 0.008), and *PTEN* (*p* < 0.001) genes in AA compared to DA, and a significantly higher level of the *EGFR* (*p* = 0.020) and *PIK3AP1* (*p* = 0.047) genes in AA compared to DA.

Further analysis using the Mann–Whitney test showed significantly higher mRNA expression of the *AKT1* (*p* = 0.002), *AKT2* (*p* = 0.030), *EGFR* (*p* = 0.001) and *PIK3AP1* (*p* < 0.001) genes, and significantly lower mRNA expression of the *AKT3* (*p*) < 0.001), *CHUK* (*p* < 0.001), *GSK3β* (*p* = 0.001) and *PTEN* (*p* < 0.001) genes in GBM versus DA. Additionally, the test showed significantly higher mRNA expression of genes *AKT1* (*p* < 0.001), *AKT2* (*p* = 0.018), and *PIK3AP1* (*p* = 0.013), and significantly lower expression of the *AKT3*, *CHUK*, *GSK3β*, and *PTEN* genes (*p* < 0.001) in GBM compared to AA. The results of mRNA expression levels were compatible with CNA findings. Table 3 and Figure 1 show the mean mRNA expression values for the examined genes.

### 3.7. Protein Expression Levels of the Examined Genes

To compare protein expression between pathohistological grades, cBioPortal protein expression data for 348 gliomas were available and downloaded. Results showed that in comparison with other proteins EGFR had the highest mean value of expression in all three grades and types. The lowest means were attributed to PTEN protein in AA, while DA and GBM showed the lowest means of GSK3β protein in comparison with other proteins (Table 4). AKT1, AKT2, AKT3, GSK3β, and EGFR expression values were higher in GBM and AA compared to DA (Table 4). Although EGFR expression values were higher and PTEN lower in AA than in DA, this difference was not significant, probably due to the relatively small number of DA samples. Additionally, no significant difference was found in EGFR protein expression between DA and GBM, although the mean of EGFR expression in GBM was higher.

### 3.8. Methylation Patterns of Examined Genes

**Figure 2:**
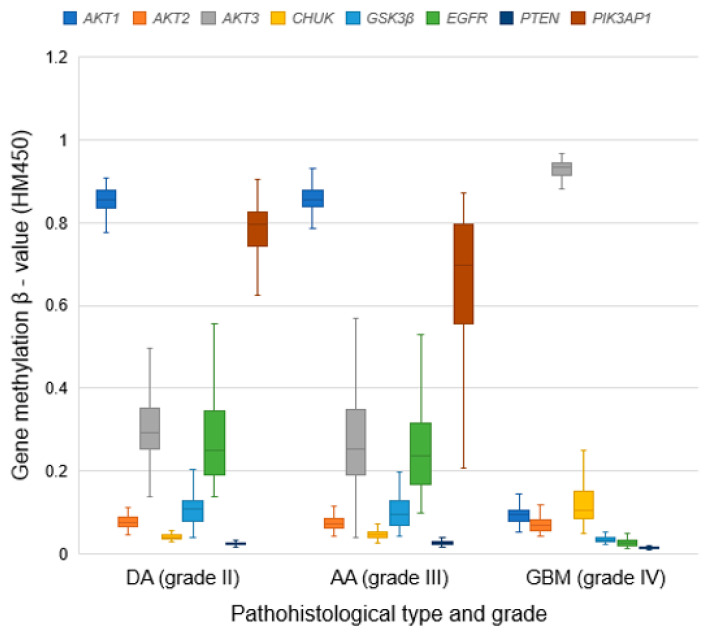
Distribution of gene methylation β-values (HM450) within each pathohistological type and grade of glioma. DA—diffuse astrocytoma; AA—anaplastic astrocytoma; GBM—glioblastoma multiforme.

The mean methylation values for the examined genes are shown in Table 5 and Figure 2. The methylation values of the *AKT3* gene were the highest in GBM, while *AKT1* showed the highest values in DA and AA. In the total sample, *PTEN* had the lowest methylation values across all grades, while the *AKT3* gene showed the highest levels of methylation (Figure 2). The Kruskal–Wallis test confirmed a significant difference in methylation of all examined genes between glioma types (*p* < 0.001 for *AKT1*, *AKT3*, *CHUK*, *GSK3β*, *EGFR*, *PTEN*, *PIK3AP1*; *p* = 0.032 for *AKT2*). Using the Mann–Whitney test, we found that methylations of the *AKT3* (*p* = 0.015) and *PIK3AP1* (*p* < 0.001) genes were significantly higher in DA in comparison with AA, while those of the *CHUK* (*p* < 0.001) and *PTEN* (*p* = 0.008) genes were significantly higher in AA compared to DA. Further analysis confirmed significantly higher methylation of the *AKT1* (*p* < 0.001), *AKT2* (*p* = 0.047), *GSK3β* (*p* < 0.001), *PTEN* (*p* < 0.001) and *EGFR* (*p* < 0.001) genes in AA compared to GBM. Contrary to that, the *AKT3* (*p* < 0.001) and *CHUK* (*p* < 0.001) genes had significantly higher methylation in GBM compared to AA. Methylation of the *AKT1*, *GSK3β*, *EGFR*, *PTEN* (*p* < 0.001), and *AKT2* (*p* = 0.018) genes was significantly lower in GBM, while the *AKT3* and *CHUK* (*p* < 0.001) genes were significantly more methylated in GBM versus DA. These results suggest a potentially significant role of methylation in regulating *AKT1*, *AKT3*, *CHUK*, *EGFR*, and *PIK3AP1* gene expression.

### 3.9. Correlation between CNA and mRNA Expression Across Glioma Types

Spearman’s test showed a significant positive correlation between mRNA expression and continuous CNA values in AA and GBM for the *AKT1*, *AKT2*, *CHUK*, *EGFR*, and *PTEN* genes (*p* < 0.001). The *EGFR* and *PTEN* genes showed a positive correlation between CNA and mRNA expression in DA (*p* < 0.05), and the *GSK3β* and *PIK3AP1* genes showed a significant positive correlation in GBM (*p* < 0.001). It is important to note that, in general, the strength of the correlation between CNA and mRNA expression also increased with the increasing grade (Table 6). These findings suggest that at higher grades, the biological aggressiveness of tumors is more dependent on CNA. Furthermore, deletions were positively correlated to lower mRNA transcript levels, while genes predominantly showing amplifications had significantly more transcripts of the amplified genes.

### 3.10. Correlation between Mezthylation and mRNA Expression Across Glioma Types

Spearman’s test established a significant negative correlation between methylation and mRNA expression of the *GSK3β*, *EGFR*, and *PIK3AP1* genes in DA (*p* < 0.05), the *AKT1* (*p* < 0.05), *AKT2*, *AKT3*, *GSK3β*, *EGFR*, and *PTEN* (*p* < 0.001) genes in AA and the *EGFR* and *PTEN* genes in GBM (*p* < 0.05). Furthermore, by comparing Spearman’s correlation coefficient (r) (Table 6), methylation in lower grades showed a stronger negative correlation with mRNA expression than the correlation between mRNA and CNA. In DA, five genes (*AKT1*, *AKT3*, *GSK3β*, *EGFR*, and *PIK3AP1*) showed a stronger correlation, in AA three genes (*AKT3*, *GSK3β*, and *EGFR*), while in GBM, only *AKT3* showed a stronger correlation between mRNA and methylation in comparison to CNA. However, in GBM, the *AKT1*, *AKT2*, *GSK3β*, *EGFR*, *PIK3AP1*, *CHUK*, and *PTEN* genes showed a stronger correlation of mRNA expression with CNA compared to mRNA and methylation (Table 6). These findings suggest that in higher-grade gliomas, CNAs were more responsible for mRNA expression, while at lower grades, methylation plays a critical role in the expression of the examined proteins. All tested genes showed this pattern of regulation except *AKT3*.

### 3.11. Correlation between Protein and mRNA Expression Levels Across Glioma Types

**Figure 3:**
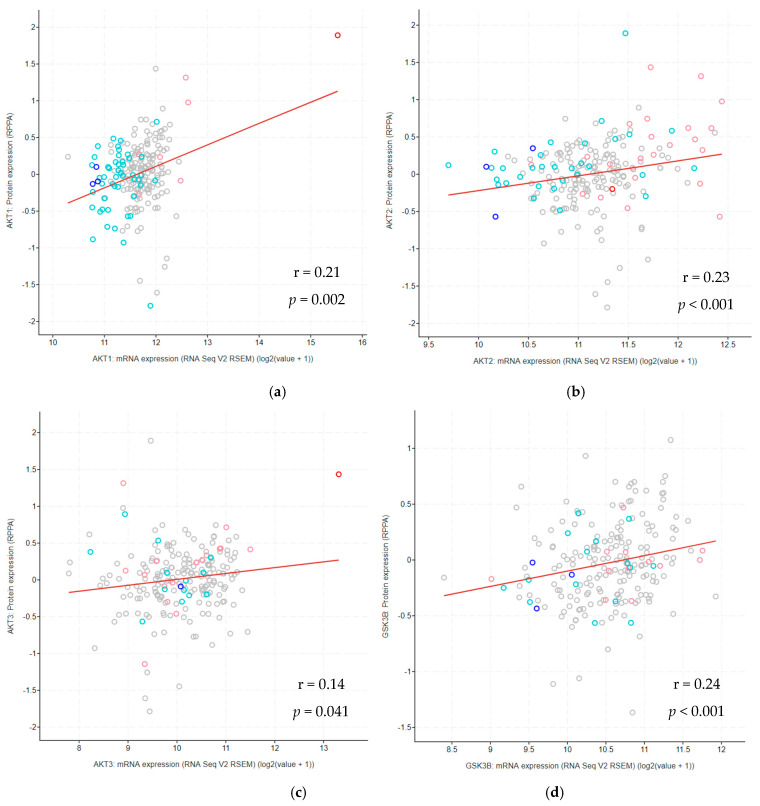

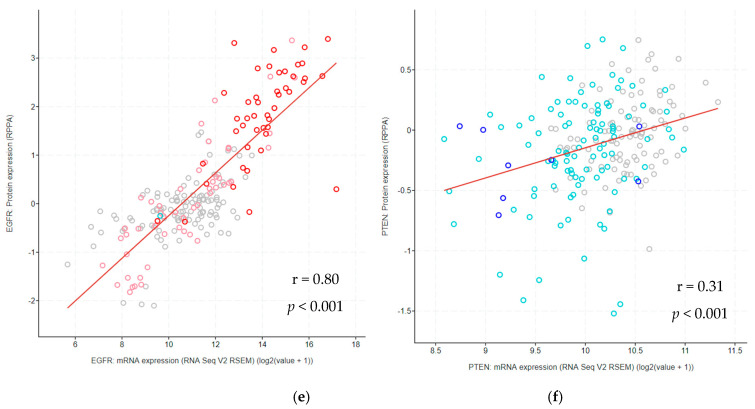
Correlation of mRNA transcription with protein expression of all diffuse gliomas samples together. The protein expression dependency on mRNA transcription has been shown for the proteins AKT1 (**a**), AKT2 (**b**), AKT3 (**c**), GSK3β (**d**), EGFR (**e**) and PTEN (**f**). Additionally, the figure shows CNA: blue dots—homozygous deletions, turquoise dots—hemizygous deletions, pink dots—amplification, red dots—multiple amplification, gray dots—samples without CNA. Samples with amplifications show increased mRNA expression, while samples with deletions show decreased transcription. Red lines represent regression lines. r—Spearman’s correlation coefficient.

Generally, the levels of mRNA expression were accompanied by protein upregulation. Spearman’s test showed a significant positive correlation between mRNA and protein expression for genes *EGFR* (*p* < 0.001) and *PTEN* (*p* < 0.001) in DA, *AKT1*, *AKT2*, and *GSK3β* (*p* < 0.05), and *EGFR* and *PTEN* (*p* < 0.001) in AA, and *AKT2* (*p* = 0.031), *GSK3β* (*p* = 0.025) and *EGFR* (*p* < 0.001) in GBM (Table 6). As shown in Table 6, the *EGFR* gene’s mRNA expression showed the strongest correlation with its protein expression in all three pathohistological types of gliomas. Moreover, looking at our total sample, all examined proteins had a statistically significant correlation with mRNA transcription (Figure 3).

## 4. Discussion

This integrative in silico study demonstrated the frequency of gene changes and their association with transcriptional, epigenetic, and protein expression levels for *AKT1*, *AKT2*, *AKT3*, *CHUK*, *GSK3β*, *EGFR*, *PTEN*, and *PIK3AP1* genes in different types and grades of diffuse brain gliomas. Our investigation on 751 gliomas showed genetic changes of *PTEN* in 76%, *PIK3AP1* and *CHUK* in 75%, and *EGFR* in 74% of the total samples. These results show a very high association level of these genes with the complex regulation of the PI3K-AKT-mTOR pathway. Other tested genes were less frequently altered—*AKT2* in 39% of samples, *AKT1* in 32% of samples, *AKT3* in 19% of samples, while *GSK3β* showed the lowest frequency of 18% of total samples. The frequency of genetic changes varied between grades, and far more CNA were attributed to GBM when compared to lower grades. When focusing on the most severe grade, a collective frequency for genetic changes of genes *EGFR*, *PIK3AP1*, and *PTEN* amount to 89%, while *CHUK* was changed in 88% and *AKT2* in 43% of GBM samples. Such a high frequency of changes at the genetic level indicates the important role of the AKT pathway in the tumorigenesis of brain gliomas [33]. Our results also showed differences in the roles of individual genes during early and late events of diffuse glioma evolution. Data on CNA show that genes *AKT2*, *AKT3*, *CHUK*, *EGFR*, *PIK3AP1*, and *PTEN* show an increase in copy number alterations with the increased glioma grade. Statistical analysis showed a significant difference in the continuous CNA number of *AKT2*, *AKT3*, *CHUK*, *EGFR*, *PIK3AP1*, and *PTEN* within different grades of diffuse gliomas (Kruskal–Wallis, *p* < 0.001). Such data suggest that these genes have a role in the progression of higher glioma grades and biological aggression. However, *AKT1* and *GSK3β* genes did not show differences in the continuous number of CNAs between grades (*p* > 0.05), which might indicate that *AKT1* and *GSK3β* play roles in the initiation of diffuse glioma formation.

The medical significance of protein kinase B (AKT) and its associated signaling pathways is evidenced by numerous studies confirming AKT activation in various types of cancer, including kidney cancer [34], papillary thyroid cancer [35], nasopharyngeal cancer [36], squamous cell carcinoma of the esophagus [37], lung cancer [38], and advanced breast cancer [39]. The genes encoding these kinases showed differences in genetic, epigenetic and the expression status of different isoforms: AKT1, AKT2, and AKT3. The present investigation showed that the *AKT1* gene was changed in 15% of DA, 30% of AA and 34% of GBM, while *AKT2* was changed in 11% of DA, 33% of AA and 43% of GBM. In contrast, *AKT3* showed CNA in only 2% of DA, 19% of AA, and 22% of GBM. Hemizygous deletions were the most common CNA found in the *AKT1* gene (26%), while amplifications were the most common CNA for *AKT3* (13%) and *AKT2* genes (26%). Our further results on methylation patterns show reduced methylation of the *AKT1* and *AKT2* genes in GBM compared to lower grades (*p* < 0.05), while the *AKT3* gene was methylated significantly higher in GBM (*p* < 0.001). These data tell us about the important role of methylation that complements genetic changes and contributes to high *AKT1*, *AKT2* expression and decreased *AKT3* expression. It is speculated that decreased *AKT3* expression improves cell dedifferentiation and tumor progression [40,41]. A significant decrease in methylation was observed for the oncogene *AKT1* (*p* < 0.001) in grade IV in comparison with grades II and III. This was accompanied by a significant increase in its mRNA expression in grade IV (*p* = 0.002, grade II; *p* < 0.001, grade III). The results of reduced methylation that enabled enhanced mRNA expression, which were significantly associated with grade IV, suggest an important oncogenic role of the *AKT1* gene. Amplifications of the *AKT2* gene in 33% of GBM samples with a significant reduction in methylation in grade IV relative to AA (*p* = 0.047) and DA (*p* = 0.018) resulted in a significant increase of mRNA expression within GBM in comparison with DA (*p* = 0.030) and AA (*p* = 0.018). Protein expression was also significantly positively correlated with increased mRNA expression (*p* = 0.031) due to frequent amplifications and decreased methylation. This may suggest enhanced activity of the *AKT2* oncogene, similar to *AKT1* in grade IV and its association with the biological aggressiveness of diffuse gliomas. On the other hand, *AKT3* showed a different picture; it significantly increased methylation within the GBM group when compared with lower grades (*p* < 0.001), which was accompanied by significantly lower mRNA expression in grade IV (*p* < 0.001). The decrease in *AKT3* gene transcription suggests its action as a tumor suppressor. Data reported in another study [2] state that approximately 70% of GBM shows enhanced activation of one of the protein kinase B (AKT) isoforms, which coincides with our results. Western blot analysis of different AKT isoforms showed that in 5/5 samples, *AKT2* was active/phosphorylated, and its mRNA expression was positively correlated with grades, while *AKT1* and *AKT3* were active in 3/5 samples [41]. The same study hypothesized that decreased *AKT3* expression leads to the dedifferentiation of cells that acquire stem-like properties and are responsible for GBM progression [41]. Recent research has confirmed this hypothesis by showing that elevated *AKT3* expression may delay glioblastoma progression [2,42]. Our results confirm reported investigations and show that *AKT1* and *AKT2* act as promoters of glioma tumorigenesis, while *AKT3* shows a tumor-suppressor role.

The findings on the *CHUK* gene showed a high proportion of CNA, of which 8% were in grade II (DA), 49% in AA, and 88% in GBM. Despite the lack of literature data on the behavior of *CHUK* in gliomas, studies on nasopharyngeal carcinomas show decreased staining of CHUK protein in undifferentiated nasopharyngeal cancers in comparison with differentiated ones [43]. Elevated mRNA levels were associated with longer survival in the same study. Such observations are in line with our results in which the largest number of hemizygous deletions of *CHUK* were found in the grade with the worst prognosis (88% of GBM). DA, which has a better prognosis, had significantly higher expression levels of *CHUK* mRNA in comparison to AA (*p* = 0.008) and GBM (*p* < 0.001). A high percentage of hemizygous deletions (74% of the total sample) of the *CHUK* gene and increased methylation, which was significantly higher in GBM compared to lower grades (*p* < 0.001), could explain lower mRNA expression in GBM (*p* < 0.001) and significantly lower mRNA expression in AA compared to DA (*p* = 0.008). The results suggest that deletions and increased methylation of this tumor suppressor play a role in diffuse glioma progression.

*GSK3β* harbored CNA in 11% of DA, 10% of AA, and 21% of GBM. Although our results showed a relatively low proportion of CNA, previous research on human glioma cell culture showed that *GSK3β* inactivation by point mutations and phosphorylation existed in 67% of glioma samples [44]. These results indicate that *GSK3β* in tumorigenesis is largely regulated by activation/inactivation via a phosphorylation mechanism and, to a lesser extent, by CNA. In the same study [44], Zhao et al. showed that the increased expression of the active form of *GSK3β* prevents angiogenesis and tumor growth. Our results show reduced mRNA expression in GBM relative to DA (*p* = 0.001) and AA (*p* < 0.001). Hemizygous deletions and amplifications of the *GSK3β* gene were each found in 9% of samples. Despite significantly lower gene methylation within GBM in comparison with DA and AA (*p* < 0.001), the integrative analysis showed a decrease in mRNA expression within GBM compared to DA (*p* = 0.001) and AA (*p* < 0.001). Based on these results, we conclude that the mRNA expression of GSK3β in GBM is not affected by gene methylation.

The proportion of CNA of the *EGFR* gene differed significantly between the three pathohistological types. DA had CNA in only 8% of samples, and 41% in AA, while GBM showed a double proportion of CNA (89%) compared to grade III. In our study, 43.6% of GBM showed amplifications and 44.1% had multiple amplifications. Other studies are in concordance with our results and indicate a high frequency of *EGFR* gene amplification in 57.4% of glioblastomas [45]. Other reports with 45% of glioblastomas having *EGFR* gene amplification are also consistent with our data [2]. We also showed essential changes at the epigenetic level of the *EGFR* gene, and significantly reduced methylation of the *EGFR* gene in GBM compared to DA and AA (*p* < 0.001). Elevated mRNA expression in GBM was also demonstrated relative to DA (*p* < 0.001), and protein expression was positively correlated with mRNA expression at all three grades (*p* < 0.001). Amplification and multiple amplification of *EGFR* in 37% and 36% of samples, respectively, as well as significantly reduced methylation of this oncogene in GBM (*p* < 0.001), is accompanied by its increased mRNA expression (*p* < 0.001). Additionally, a significant increase in mRNA expression in AA in comparison with DA (Mann–Whitney, *p* < 0.02) was established. This result shows the important role of *EGFR* in the activation of the PI3K-AKT-mTOR pathway and its involvement in the progression of diffuse gliomas.

Our next candidate, the tumor suppressor *PTEN*, showed changes in 8% of DA, 50% of AA, and 89% of GBM. Although the other studies show that about 36% [2] and 34.3% [45] of GBM show homozygous deletions, our analysis found only 10% of samples with homozygous deletions, while hemizygous deletions were found in 79% of GBM. Consistent with our results are the outcomes of immunohistochemical analysis of GBM that showed significantly lower expression of *PTEN* (*p* < 0.05) in tumor tissue compared to the surrounding healthy brain tissue [46]. In addition to decreased methylation of the *PTEN* gene in GBM relative to lower grades, CNA within the GBM group was also strongly positively correlated with mRNA expression (*p* < 0.001), and samples with homozygous deletions showed the lowest mRNA expression values.

The *PIK3AP1* gene, coding for TLR (toll-like receptor) adapter protein that links the AKT-mTOR signaling pathway via the PI3K kinase (phosphoinositide 3-kinase) [47], was hit by CNA in 8% of DA, 49% of AA, and 89% of GBM samples. Although the role of this protein in brain gliomas is still insufficiently investigated, recent research performed on cell culture of macrophages and B lymphocytes shows hyperphosphorylation of the PIK3AP1 protein, which enables the activation of PI3K-AKT signaling [48]. Significantly higher mRNA expression in GBM was confirmed compared to DA (*p* < 0.001) and AA (*p* = 0.013), suggesting a possible role in PI3K pathway signal transduction in higher grades. Our results show that AA has significantly reduced methylation of *PIK3AP1* compared to DA (*p* < 0.001), and we can assume that reduced methylation in GBM might affect the enhanced expression, although data on *PIK3AP1* methylation in GBM were not available.

Our previous studies on primary brain tumors revealed the importance of the molecular profile in tumorigenesis and progression of intracranial tumors [49], as well as the recognizable molecular behavior of astrocytomas of different grades [50]. Such results suggest the importance of upgrading diagnostic and prognostic classification that allows understanding the behavior of tumor cells that have adapted to survive in a highly competitive brain environment. Due to the complexity of PI3K-AKT-mTOR signaling regulation, further research is needed to fully understand this pathway and its role in gliomas.

## 5. Conclusions

Our in silico analysis shows distinct roles of EGFR-PI3K-AKT-mTOR pathway participants in the tumorigenesis of diffuse brain gliomas. The results of this integrative cBioPortal analysis suggest that genes *AKT3*, *CHUK* and *PTEN* behave like tumor suppressors, while *AKT1*, *AKT2*, *EGFR*, and *PIK3AP1* show oncogenic behavior and are involved in enhanced activity of the EGFR-PI3K-AKT-mTOR signaling pathway. On the other hand, *GSK3β* showed inconsistent behavior, with increased mRNA and protein expression in AA and decreased mRNA expression in GBM, followed by reduced protein expression. Our findings contribute to molecular knowledge of the differences between pathohistological types and grades of diffuse brain gliomas. We have demonstrated that CNAs are specifically associated with a decrease or increase in transcript levels. Furthermore, methylation profiles were consistent with the type of observed genetic changes as well as with protein levels. Additionally, we specified changes associated with higher grades that contribute to progression, as well as those that are constantly appearing across grades and can be characterized as early events. Our results have the potential for improvement in diagnostics and tailored therapies.

## Figures and Tables

**Table 1:** Age and sex of patients according to pathohistological type and grade of diffuse gliomas.

PHT and Grade	Age/Years		Sex	
	Median	IQR	M	F
DA (grade II)	34.5 *^,^***	12	36	26
AA (grade III)	44.0 *^,^**	21	70	59
GBM (grade IV)	59.0 **^,^***	18	340	220
Total	55.0	22	446	305

PHT—pathohistological type; DA—diffuse astrocytoma; AA—anaplastic astrocytoma; GBM—glioblastoma multiforme; M—male; F—female; IQR—interquartile range; *—DA vs. AA (*p* < 0.001); **—AA vs. GBM (*p* < 0.001); ***—DA vs. GBM (*p* < 0.001).

**Table 2:** Percentages of samples with CNA within each pathohistological type of diffuse gliomas.

GENE	PHT	N	CNA/%				
			HOMDEL	HETLOSS	DIPLOID	GAIN	AMP
*AKT1*	DA	62	0	15	85	0	0
	AA	129	2	26	70	2	0
	GBM	560	0	27	66	6	1
*AKT2* **^,^***	DA	62	3	6	89	0	2
	AA	129	2	20	67	10	1
	GBM	560	0	10	57	33	1
*AKT3* **^,^***	DA	62	0	0	98	2	0
	AA	129	1	7	81	10	1
	GBM	560	0	4	78	15	2
*CHUK* *^,^**^,^***	DA	62	0	8	92	0	0
	AA	129	1	47	51	1	0
	GBM	560	0	88	12	0	0
*GSK3β*	DA	62	5	5	89	2	0
	AA	129	0	6	90	4	0
	GBM	560	0	9	79	11	0
*EGFR* *^,^**^,^***	DA	62	0	2	92	5	2
	AA	129	0	1	59	22	19
	GBM	560	0	1	11	44	44
*PTEN* *^,^**^,^***	DA	62	0	8	92	0	0
	AA	129	4	64	50	0	0
	GBM	560	10	79	11	0	0
*PIK3AP1* *^,^**^,^***	DA	62	0	8	92	0	0
	AA	129	2	47	51	0	1
	GBM	560	1	88	11	0	0

DA—diffuse astrocytoma; AA—anaplastic astrocytoma; GBM—glioblastoma multiforme; PHT—pathohistological type; HOMDEL—homozygous deletion; HETLOSS—hemizygous deletion; D—diploid; GAIN—amplification; AMP—multiple amplification; N—number of samples; *—DA vs. AA (*p* < 0.001); **—AA vs. GBM (*p* < 0.001); ***—DA vs. GBM (*p* < 0.001).

**Table 3:** mRNA expression (RSEM) values of the examined genes within each pathohistological type and grade of glioma.

PHT	N	mRNA Expression	GENE							
			*AKT1*	*AKT2*	*AKT3*	*CHUK*	*GSK3β*	*EGFR*	*PTEN*	*PIK3AP1*
DA grade II	62	M	3123.58 *	2245.96 *	1450.79 *	501.64 *	1530.27 *	1901.81 *	1403.18 *	311.42 *
		IQR	712.24	785.86	632.17	140.73	594.58	2082.12	337.54	289.02
AA grade III	129	M	3213.98 *	2179.75 *	1174.06 *	442.53 *	1659.60 *	2612.05 *	1212.54 *	364.66 *
		IQR	1076.78	853.40	796.81	147.41	905.05	5112.99	464.74	362.59
GBM grade IV	140	M	3737.21 *	2394.95 *	693.48 *	383.62*	1215.72 *	4179.58 *	952.07*	536.57 *
		IQR	1778.33	1223.51	414.06	128.15	693.82	14,797.33	448.76	517.91

PHT—pathohistological type; DA—diffuse astrocytoma; AA—anaplastic astrocytoma; GBM—glioblastoma multiforme; N—number of samples; M—median; IQR—interquartile range; *—*p* < 0.05.

**Table 4:** Values of protein expression (RPPA) of the examined genes within each pathohistological type and grade of glioma.

PHT	N	Protein Expression	PROTEIN			
			*AKT1/AKT2/AKT3*	*GSK3β*	*EGFR*	*PTEN*
DA grade II	46	M	−0.024	−0.160	0.042	−0.055
		IQR	0.388	0.531	0.468	0.467
AA grade III	99	M	0.082	−0.051	0.023	−0.162
		IQR	0.432	0.435	1.276	0.421
GBM grade IV	213	M	0.073	0.0004	−0.116	0.018
		IQR	0.605	0.499	2.691	0.456

PHT—pathohistological type; DA—diffuse astrocytoma; AA—anaplastic astrocytoma; GBM—glioblastoma multiforme; N—number of samples; M—median; IQR—interquartile range.

**Table 5:** Methylation β-values (HM450) of the examined genes within each pathohistological type and grade of diffuse gliomas.

PHT	N	Methylation	GENE							
			*AKT1*	*AKT2*	*AKT3*	*CHUK*	*GSK3β*	*EGFR*	*PTEN*	*PIK3AP1*
DA grade II	62	M	0.856 *	0.075 *	0.294 *	0.040 *	0.108 *	0.250 *	0.025 *	0.796 *
		IQR	0.044	0.024	0.099	0.010	0.049	0.155	0.005	0.083
AA grade III	129	M	0.856 *	0.073 *	0.252 *	0.045 *	0.095 *	0.235 *	0.026 *	0.707 *
		IQR	0.039	0.022	0.154	0.013	0.060	0.147	0.006	0.238
GBM grade IV	123	M	0.096 *	0.068 *	0.934 *	0.105 *	0.032 *	0.025 *	0.014 *	-
		IQR	0.027	0.024	0.029	0.067	0.010	0.012	0.003	-

PHT—pathohistological type; DA—diffuse astrocytoma; AA—anaplastic astrocytoma; GBM—glioblastoma multiforme; N—number of samples; HM450—Human Methylation 450 Infinium array; M—median; IQR—interquartile range; *—*p* < 0.05.

**Table 6:** Correlation of CNA, methylation and protein expression with mRNA expression in three pathohistological types of glioma.

PHT	GENE	CNA vs. mRNA Expression	Methylation vs. mRNA Expression	Protein Expression vs. mRNA Expression
		r	r	r
DA grade II	*AKT1*	0.10	−0.15	0.10
	*AKT2*	0.21	−0.10	0.12
	*AKT3*	−0.06	−0.22	0.27
	*CHUK*	0.22	−0.16	-
	*GSK3β*	0.17	−0.29 *	0.16
	*EGFR*	0.25 *	−0.32 *	0.55 **
	*PTEN*	0.28 *	−0.02	0.46 *
	*PIK3AP1*	0.11	−0.28 *	-
AA grade III	*AKT1*	0.50 **	−0.26 *	0.32 *
	*AKT2*	0.49 **	−0.42 **	0.29 *
	*AKT3*	−0.03	−0.45 **	0.01
	*CHUK*	0.69 **	−0.45 **	-
	*GSK3β*	0.15	−0.36 **	0.23 *
	*EGFR*	0.48 **	−0.49 **	0.73 **
	*PTEN*	0.59 **	−0.32 **	0.37 **
	*PIK3AP1*	0.14	−0.12	-
GBM grade IV	*AKT1*	0.61 **	−0.06	0.24
	*AKT2*	0.47 **	−0.30	0.27 *
	*AKT3*	0.05	0.34 *	0.15
	*CHUK*	0.56 **	0.05	-
	*GSK3β*	0.30 **	0.09	0.28 *
	*EGFR*	0.79 **	−0.44 *	0.89 **
	*PTEN*	0.56 **	−0.40 **	0.19
	*PIK3AP1*	0.49 **	-	-

PHT—pathohistological type; DA—diffuse astrocytoma; AA—anaplastic astrocytoma; GBM—glioblastoma multiforme; r—Spearman’s correlation coefficient; *—*p* < 0.05; **—*p* < 0.001.

## Data Availability

Data supporting reported results are contained within the article. The data presented in this study are available on request from the corresponding author. Generated cBioPortal virtual study data are available online at https://www.cbioportal.org/study?id=609e74e2e4b015b63e9eb2d6.

## Supplementary Materials

Virtual study data are available online at https://www.cbioportal.org/study?id=609e74e2e4b015b63e9eb2d6.
